# Sensory Processing Disorder in Children—Description of the Phenomenon and Practical Procedures

**DOI:** 10.3390/jcm14124105

**Published:** 2025-06-10

**Authors:** Izabela Maćkowiak, Julia Ciesielska, Monika Ruszczyk, Justyna Opydo-Szymaczek, Natalia Torlińska-Walkowiak

**Affiliations:** 1 Department of Orthodontics and Temporomandibular Disorders, Poznan University of Medical Sciences, 70 Bukowska Street, 60-812 Poznan, Poland; 2 Department of Pediatric Dentistry, The Student Scientific Society of Poznan University of Medical Sciences, 60-812 Poznan, Poland; 3 Department of Pediatric Dentistry, Poznan University of Medical Sciences, 70 Bukowska Street, 60-812 Poznan, Poland

**Keywords:** sensory processing disorder, hyposensitivity, hypersensitivity, healthcare professionals, dental treatment, surgical office, sensory integration disorder

## Abstract

Sensory processing disorder (SPD) involves difficulties in receiving and responding to sensory information from the environment. Their development is influenced by various factors during the perinatal period and early childhood. Children with sensory integration disorders often struggle with everyday situations and stress, typically experiencing either sensory hypersensitivity or hyposensitivity. Their visit to a doctor’s office presents challenges for both the child and the medical practitioner, requiring active cooperation from parents. This review aims to analyze the challenges faced by clinicians in managing pediatric patients with SPD during healthcare visits, with a specific focus on dental settings and to propose effective communication and management strategies. Proper preparation of the child before the visit and the use of appropriate communication techniques during the appointment play a crucial role in ensuring a smooth experience. Strategies such as minimizing visual and auditory stimuli, applying recommended touch techniques, and opting for the least invasive treatment methods can help create a child-friendly environment and improve the overall quality of care.

## 1. Introduction

Sensory integration processing is a term introduced primarily by Ayres and stands for the sensory–motor processes that enable organisms to move, act intentionally, interact in a social environment and respond appropriately to various stimuli [1].

Sensory processing disorder (SPD) is a neurological condition in which the brain has difficulty receiving and responding appropriately to information from the senses. Within SPD, some patterns of response are commonly observed: sensory over-responsivity, where individuals have an exaggerated response to sensory stimuli (e.g., distress from normal levels of sound, light, or touch), sensory under-responsivity, where individuals show a reduced or delayed response to stimuli (e.g., failing to notice intense smells or tactile sensations) or craving for sensory input [2,3]. Both hypersensitivity and hyposensitivity can significantly impact a child’s daily functioning (for example, the texture, taste, smell or appearance of food may trigger a strong aversive reaction), as well as learning and participation in social or clinical settings [4,5].

Recent studies indicate that 15.9% of the pediatric population in Europe and 5% in the USA present with the severe sensory processing disorder (SPD) [6,7]. Studies of children with SPD give evidence of psycho-physiological impairments in both sympathetic and parasympathetic nervous system reactions [1]. However, it was demonstrated that sensory processing dysfunction could be diagnosed in neurotypical children who exhibit atypical reactions to some stimuli [3,8].

One of the known risk factors for disorganized sensory integration processes in the nervous system is known to be premature birth, along with related complications, such as sepsis, postnatal steroids use, assisted delivery, gestational or birth-related injury/illness and developmental problems [9] (Table 1).

Numerous studies have investigated the impact of premature birth on children’s daily routines and activities [10,11,12]. Appropriate sensory stimulation after birth is essential for forming neural connections in the brain. Prematurely born children do not experience sufficient tactile–vestibular stimulation resulting from the maternal movement and are instead exposed to intense light and noise stimuli that do not occur in the intrauterine environment, which may contribute to sensory perception disorders [9].

Various methods are used to diagnose SPD, including neuroimaging techniques to assess attention-related challenges and specific caregivers-administered questionnaires [6,7]. One of them is SP-2 (Sensory Profile 2) which is a questionnaire to evaluate sensory processing patterns (seeking, avoiding, sensitivity, and registration) and other areas influenced by SPD: auditory, visual, oral, movement, social behavior [6,13]. 

This review aims to analyze the challenges faced by clinicians in managing pediatric patients with SPD during healthcare visits, with a specific focus on dental settings, and to propose effective communication and management strategies.

Forty one articles published since the year 2010 were qualified for the study from the following databases: Pubmed, GoogleScholar, and Scopus. The inclusion criteria comprise the following key words: sensory processing disorder, integration processing, children, dental office, medical examination, non-verbal communication.

### Challenges in the Cooperation Between Medical Professionals and Patients with Sensory Processing Disorders

The literature identifies various barriers that individuals with developmental disorders—particularly those related to sensory integration—face when seeking professional medical care. An inadequately trained physician may misinterpret a patient’s behavior [14,22,23,24], especially if their education does not provide adequate preparation for working with patients with special needs [25,26].

Lane et al. [1] emphasize that modifying the environment where a medical visit takes place is crucial for supporting children with sensory processing disorders. These changes need to be individual, tailored to the child’s requirements, and can significantly reduce stress during the visit [27]. Some non-sensory interventions, such as a task-based approach (e.g., recreational exercises) can help children with SPD improve self-regulation and behavioral control during medical procedures [27].

Multisensory environments are also gaining popularity as a non-pharmacological approach to managing anxiety and stress in children with SPD. However, the exact mechanisms of their effectiveness remain unclear [28]. Adequately adapting the child’s daily functioning, pointing out the appropriate ways of self-regulation, and reducing anxiety and stress can significantly improve the child’s ability to cope with stressful situations, such as visits to a dentist or other specialists working in the head and neck region [14].

Difficulties in adapting to a visit are observed during most medical appointments but are particularly noticeable in the emergency department and dental clinics [15,24,29]. Unfortunately, negative reactions may accompany children from the very moment they enter the building and accompany them through subsequent stages of treatment [30].

According to Kim et al. most parents of children with Autism Spectrum Disorder (ASD) rate their child’s experience at the dental office as “negative” [14]. This is not surprising, as research indicates that up to 97% of children with ASD exhibit atypical sensory processing patterns [31]. Furthermore, sensory challenges are very common in children with disorders, such as ADHD, Fragile X syndrome, Down syndrome, intellectual disability, and cerebral palsy [16,17,18].

Many patients’ oral health problems are related to harmful habits such as bruxism, tongue thrusting, and difficulties with cleaning their teeth thoroughly. These risk factors interact with each other and lead to caries, malocclusion, gingivitis, and periodontal disease, which are often detected at more advanced stages when hypersensitivity makes daily oral hygiene difficult to maintain [26,32,33]. For other specialists, such as pediatric ENT doctors, children with SPD experience common conditions like otitis media but may report symptoms later due to communication difficulties, leading to a more severe disease course, increased hospitalizations, and more frequent surgical interventions [34].

The responses of children with SPD to the clinical environment can vary significantly depending on their sensory profile. Children with sensory hypersensitivity tend to have exaggerated responses to ordinary sensory stimuli. For example, the sound of a dental drill or the bright light of an examination lamp can be overwhelming, leading to distress or behavioral resistance. Excessive sensitivity to movement can result in anxiety due to sudden chair position changes or headrest adjustments [30,32]. Reluctance to touch triggers the need to push the doctor’s hands away during the examination and difficulty keeping the mouth open. Sensitive eyesight can cause tearing and squinting when exposed to the light of the unit lamp. In addition, the sound of dental equipment or metal tools placed on a tray, a prolonged visit, and the mere sight of the assistant and doctor wearing face masks can cause anxiety [30,32]. Similarly, the intense tastes and smells of medications and dental materials can trigger an increased vomiting reflex and be overwhelming for a patient with sensory integration disorders [30,32].

In contrast, sensory hyposensitivity involves a reduced or delayed response to sensory input. These children may not notice sensations that others do, such as pain, temperature, or loud sounds. As a result, they may seek out additional or more intense sensory experiences to meet their neurological needs. This can manifest as a constant need to touch objects, a preference for loud music, excessive movement such as spinning or jumping, or a high tolerance for pain. Children with hyposensitivity might appear inattentive, withdrawn, or unresponsive to their environment, and they may engage in sensory-seeking behaviors to compensate for the lack of sensory input they perceive [2,35].

Given this range of sensory profiles, medical professionals may encounter a wide spectrum of behaviors during visits, including the following:

- kicking, hitting, pushing, biting,
- tantrums and screaming,
- crying,
- running away from the chair and hiding,
- avoiding eye contact and hiding your face in your hands [32].

This diversity of responses highlights the importance of individualized care strategies that account for each child’s unique sensory needs.

## 2. Clinical Management

Effective clinical management for children with SPD requires an understanding of the specific sensory challenges each child faces.

For children with sensory hypersensitivity, even routine clinical procedures can be overwhelming, often triggering distress or avoidance behaviors. To reduce sensory overload, it is important to create a calming and predictable clinical environment. Effective strategies include using dimmer, softer lighting, minimizing sudden or unexpected physical contact, and selecting unscented or lightly scented dental materials to reduce the impact of strong smells. Examination chair should be set in the desired position in advance so that the child can only sit/lie down on it and avoid position changes during the procedure [30,32,36]. Excessive light stimuli can be overwhelming for children. Therefore, using very bright light is not advised, and the lamp from the dental unit should not shine directly into the child’s eyes. The use of sunglasses is also worth suggesting as a simple method to prevent the child from overstimulating. Any flashing lights should be covered or switched off [15,19,29].

Doctors and medical staff should not use perfumes or scented body cosmetics. It is also advised to give up perfumed candles and other air refresheners [20]. If possible, cleaning products used in the office should also be unscented, as all these odors may trigger an excessive reaction in some children with SPD. It is also worth paying attention to the taste, smell and texture of the pastes and gloves used in the office, because some of them may be irritating to the child. Offering children a choice of flavored materials (e.g., fluoride varnishes oral polishing pastes) helps to avoid unpleasant sensory reactions [30].

Unnecessary touch must be avoided, and the child should be informed before touching his or her face. A more decisive and strong touch will work better than a gentle one [19]. To improve the child’s comfort during the procedure, he or she should also be allowed to rinse his or her mouth frequently [32,36].

All unnecessary sounds should be eliminated, and the office should be quiet and isolated from the outside sounds. If possible, the use of dental handpieces should be limited, and the materials should be mixed manually. Using encapsulated materials prepared in a capsule mixer generates additional unnecessary sound stimuli. Children can use headphones to block out the surroundings or listen to their own music. The use of white noise devices may be helpful, but this strategy will not always work, so it must be agreed with the parent in advance. It is crucial to avoid loud and unexpected sounds that could accompany, e.g., tools hitting the tray—an additional towel or napkin can be used to reduce it [24,30,32].

The sensory-adapted dental environment (SADE), featured by Cermack [19,21], becomes increasingly popular. Certain changes are implemented in comparison to a regular dental environment. Darkening curtains should be installed on the windows and all the direct overhead lamps should be turned off. Ambient music and light may be projected into the room. Dental chair can be wrapped resembling a butterfly and its wings can provide deep sensory input (deep pressure technique) [21]. It is reported that the use of this technique is beneficial and could potentially be used in different developmental disabilities that come with sensory symptoms [14].

Gradual desensitization is another effective approach, as it allows children to slowly build tolerance to challenging stimuli over time. For example, a child who is sensitive to the sound of dental instruments might benefit from a stepwise exposure strategy, beginning with less invasive procedures and gradually progressing to more complex treatments as their tolerance improves. Research supports the use of desensitization techniques, showing that they can significantly reduce uncooperative behavior during both dental and medical procedures [19,24,32,36].

In contrast, children with sensory hyposensitivity often require more intense sensory input to stay attentive. These children may not react as strongly to tactile sensations, sounds, or movement, often seeking out more intense sensory experiences to compensate. For these children, strategies that increase sensory input can be beneficial. This might involve using brightly colored, textured objects during procedures, allowing short movement breaks, or incorporating vibration (e.g., through electric toothbrushes) to enhance their sensory awareness. These approaches can help maintain the child’s focus and reduce restlessness during clinical visits [35].

Deep pressure technique is recommended, as for some children, the added weight may have a calming effect. After consultation with the parent, with his/her consent, a heavy radiological gown can be used. A strong hug from the parent when the child is sitting in a chair on the parent’s lap may also be helpful. The doctor should also offer the possibility of bringing their own blanket or toys that calm the child [19,32,37,38]. Just as the deep pressure technique is routinely used in sensory integration therapy, the hard work technique is used both in therapy and during dental visits. During the procedure, the child could be given a toy that attracts his attention, e.g., a fidget spinner or various types of balls or squeeze masses [29,32]. Both strategies can be useful in the dentist’s office, but their use should be discussed with the parent in advance because it might not be beneficial for every child [32].

To minimize stress, parents should prepare the child well in advance of the appointment. Effective preparation may differ according to the type of stimuli that the child prefers. A variety of picture books or educational videos about visiting a doctor’s office, especially a dentist’s office, can be helpful. Viewed in good time before the visit—a week or more—they will give the child a chance, and time, to process the information and ask questions that will clear up his or her doubts [30]. Introducing electric toothbrushes in daily oral hygiene routines helps children to adapt to the sounds and vibrations of dental procedures. The sounds and vibrations emitted by the toothbrushes will help prepare the child, at least to some extent, for the auditory and tactile stimuli within the oral cavity at the dentist’s office. Pre-visit consultation between the parent and the dentist might also be helpful [14,19,30]. This allows a calm discussion about the child’s unique sensory needs [13,14,19] and helps both the parent and the dentist to prepare for the visit [32]. Engaging in physical activities (e.g., playground visits) before the appointment may help children who require intense sensory input expend excess energy [30].

Minimally invasive methods of caries treatment seem to be the most appropriate when the doctor admits children with SPD. The use of hand tools significantly reduces noise exposure that may overstimulate the child. Research indicates that in children with disabilities, the annual survival rate of fillings made using the ART technique was higher than in the case of conventional fillings. However, other effective, non-invasive caries treatment methods, such as CMCR (chemomechanical caries removal) or air abrasion, still require further validation for use in children with SPD [32,36,39]. The role of prophylaxis and maintaining oral hygiene at home is crucial to avoid the need for an invasive treatment [33].

Oral sedation, e.g., hydroxyzine or midazolam, could also be used. Guidelines for children suffering from ASD indicate the possibility of using these methods; however, since the child must cooperate with the doctor at least to some extent, they are difficult to implement. SPD is common in children with ASD and may be the reason for their lack of cooperation in the dental office. However, it would be necessary to conduct additional research to determine how the use of sedation may affect the behavior of children with SPD, and not only in children with ASD. When it is impossible to establish cooperation between the child and the doctor, the parent may consider treatment under deep sedation or general anesthesia. This procedure requires the additional presence of an anesthesiologist and is often impossible to perform on an outpatient basis [19,33,39,40,41].

## 3. Communication Techniques

Standard communication methods used by doctors have also proven effective in working with children with SPD. Positive reinforcement rewards desired behavior in a child. By using this technique, the doctor increases the likelihood of the behavior returning.

Tell–show–do is one of the most frequently used methods during dental appointments. Explaining and demonstrating procedures before performing them helps the doctor to prepare children for subsequent stimuli and shapes their behavior. Thanks to this, children with SPD know exactly what to expect at a given stage of the visit [36].

Studies have shown that distracting a child’s attention from unpleasant experiences, for example, when using VR glasses, has proven to be effective in children with ASD, which is often associated with SPD [32].

The desensitization method allows children with SPD to withstand expected stimuli. Gradual introduction of new stimuli at subsequent visits, starting with the inspection itself, adding, e.g., varnishing at the next visit, and only then proceeding with more invasive procedures, may bring positive effects [19,32,36]. According to the research, using desensitization techniques can reduce uncooperative behavior during both dental and medical procedures [24].

Simple commands work better than polite questions [36]. It should not be assumed that a child who does not communicate verbally does not understand verbal and non-verbal messages. It may also be helpful to set a specific limit when a given activity performed by the doctor will end. The patient would be given a scale of control over the dentists’ behavior during the use of stop signals. A countdown can be used in this case [19,32]. In many articles, researchers indicate that it is effective to prepare indicators that will show the child the end of the procedure. For this purpose, for example, stopwatches with a large display might be functional. Additionally, pictures could be used that will show the child how the visit will proceed and what it will include [30,32,36].

Children with SPD often avoid eye contact and focus on the interlocutor’s mouth movements. For this reason, whenever possible, the doctor should also avoid looking into the child’s eyes and should use facial protection that allows the child to see his/her mouth. However, this is not possible when the doctor and medical staff are wearing standard masks [30,36].

## 4. Conclusions

Sensory integration disorder presents significant challenges in medical and dental settings. However, a combination of communication strategies, environmental modifications, and minimally invasive treatment approaches can greatly improve the experience for these patients. Many of these techniques can be successfully implemented not only by dentists but by all healthcare professionals working with children with SPD.

This review highlights a range of practical procedures that can be implemented during medical and dental visits for children with SPD. The effectiveness of these interventions lies in their adaptability to each child’s unique sensory profile. Practical implementation requires interdisciplinary collaboration and pre-visit planning with caregivers to ensure the child’s needs are adequately accommodated.

## Figures and Tables

**Table 1:** Etiology, prevalence, and frequency of SPD.

References	Country	Etiological Factor	Reported Frequency	Diseases Connected with Integration Disorders
Galiana et al. (2022) [6]	Spain	premature birth	15.9% severe; 10.5–11.1% moderate	ADHD, Autism spectrum disorder
Gonçalves et al. (2022) [10]	Portugal		43%	Autism spectrum disorder
Eeles et al. (2013) [11]	Australia	premature birth	of the combined sample of 327 VPT children, 253 had data for the Infant/Toddler Sensory Profile at 2 years’ corrected age.	
Adams et al. (2015) [12]	USA	preterm birth	37% of preterm than full term preschoolers had elevated numbers of sensory symptoms	Williams syndrome, autism
Critz et al. (2015) [13]	USA	neurologically based problems	10–55%	Autism spectrum disorder (ASD)
Kim et al. (2019) [14]	USA	developmental disorders	46% of parents would prefer SADE for their children	Down syndrome, cerebral palsy, developmental delay, Autism spectrum disorder
Wood et al. (2019) [15]	USA		5–15%	Autism spectrum disorder (ASD), attention deficit/hyperactivity disorder (ADHD), developmental coordination disorders, children anxiety disorders
Nair et al. (2023) [16]	Finland		8.3% of 8-year-old	ASD, ADHD, Fragile X syndrome
	USA		1 out of 20 children	
	General population		5–15%	
Pavão et al. (2017) [17]	USA	white matter lesion	children with cerebral palsy had worse sensory processing compared to typical children (the power of the statistical analysis was 75%)	Cerebral palsy
Bruni et al. (2010) [18]	USA			Down syndrome, attention deficit/hyperactivity disorder (ADHD), autism spectrum disorder (ASD), Fragile X syndrome
Nelson et al. (2015) [19]	general population	“complex interplay of genetic and epigenetic factors”—as etiology of ASD	3.4–15.6%	ASD
Goodman-Scott et al. (2015) [20]	USA	genetic basis	5–17% of children	Autism spectrum disorder, attention-deficit/hyperactivity disorder (ADHD), anxiety disorders, depression, Fragile X syndrome and obsessive–compulsive disorder
Cermak et al. (2015) [21]	USA		95% of children with ASD	ASD

## Data Availability

Not applicable.
